# Evaluation of *in vitro* pharmacological activities of medicinal mushrooms in the context of dry eye disease

**DOI:** 10.3389/fphar.2025.1557359

**Published:** 2025-03-05

**Authors:** Alexander Areesanan, Andreas Wasilewicz, Sven Nicolay, Ulrike Grienke, Amy M. Zimmermann-Klemd, Judith M. Rollinger, Carsten Gründemann

**Affiliations:** ^1^ Translational Complementary Medicine, Department of Pharmaceutical Sciences, University of Basel, Basel, Switzerland; ^2^ Division of Pharmacognosy, Department of Pharmaceutical Sciences, Faculty of Life Sciences, University of Vienna, Vienna, Austria

**Keywords:** dry eye disease, antioxidant, inflammation, reactive oxygen species, proinflammatory cytokines, polypore, medicinal mushroom

## Abstract

**Introduction:**

Ethnic groups worldwide use mushrooms, particularly polypores (a group of fungi with woody fruiting bodies), to manage inflammatory conditions. In this study, the *in vitro* anti-inflammatory potential and mycochemical composition of six polypore extracts derived from the fruit bodies of *Fomes fomentarius* (L.) Fr. (FF), *Ganoderma lucidum* (Fr.) P. Karst. (GL), *Ganoderma tsugae* Murrill (GT), *Gloeophyllum odoratum* (Wulfen) Imazeki (GO), *Laricifomes officinalis* (Vill.) Kotl. and Pouzar (LO), and the sclerotium of *Inonotus obliquus* (Fr.) Pilát (IO) were analyzed for their relevance to treat dry eye disease (DED).

**Methods:**

Ethanolic extracts of the fungal materials were prepared and chemically characterized by UHPLC-ELSD/MS and TLC analyses before investigating the extracts’ cytotoxic, antioxidant, anti-inflammatory, and lipid-stimulating properties. Radical scavenging and intracellular reactive oxygen species (ROS) assays were carried out in UVB-exposed human corneal epithelial (HCE-T) and immortalized human meibomian gland epithelial (IHMGEC) cells to evaluate antioxidant capacities. To examine the influence of the extracts of the inflammatory processes, associated with DED, a secretion assay for pro-inflammatory cytokines was conducted in UVB-exposed HCE-T and LPS-stimulated monocytic THP-1 cells. The lipid droplets secreted by IHMGECs were analyzed to determine the extracts’ lipid-stimulating properties.

**Results:**

Extracts of GT, GL, GO, and IO found to have high radical scavenging abilities. They significantly reduced intracellular ROS in UVB-exposed HCE-T and iHMGEC cells. GO and GL extracts inhibited cytokine secretion in HCE-T cells even at low concentrations. All tested extracts significantly inhibited the secretion of pro-inflammatory cytokines (IP10, IL-6, IL-8, and α) in LPS-stimulated monocytic THP-1 cells.

**Conclusion:**

Several extracts of the investigated fungal materials exhibit multifaceted pharmacological *in vitro* activities. Due to low cytotoxic activity on HCE-T, iHMGEC, and THP-1 cells, extracts from GL and GO are particularly pertinent to the treatment of DED, even at low concentrations.

## 1 Introduction

Dry eye disease (DED) is a widespread multifactorial condition that affects 5%–50% of the population worldwide, especially elder women. Prevalence is steadily increasing, and the condition is rising, becoming one of the top public health concerns (Papas, 2021). DED is characterized by either hyper-evaporative tears (10% of cases), or a lack of the quality of the tear film (80% of cases), or a combination of both (10% of cases) (Rouen and White, 2018; Tsubota et al., 2017). Symptoms of DED can occur individually or in combination and include visual disturbance, grittiness, itchiness, and burning sensation. DED symptoms make it difficult for the patients to perform any tasks efficiently (Sakane et al., 2013) and thus severely impair the quality of life (QOL). Without proper treatment, symptoms tend to worsen (Baudouin et al., 2014). The total economic burdens due to reduced productivity and medical care as a result of DED has been estimated to be over $55 billion in the United States (Yu et al., 2011) and over $100 billion in China (Yang et al., 2021).

It is believed that inflammation, which is linked to hyperosmolarity and tear film instability, is the key cause of DED pathogenesis (Wei and Asbell, 2014). Unlike other mucosal surfaces in the body, the microenvironment of the ocular surface is constantly exposed to noxious insults such as pathogens, pollution, and harmful substances that can disrupt the ocular homeostasis. Such conditions severely stress the ocular surface by increasing hyperosmolarity and tear film instability, and lead to reactive oxygen species (ROS) formation, setting off a cascade of several inflammatory cytokines followed by apoptosis (Bron et al., 2017). Studies have demonstrated that DED is characterized by inflammatory cytokines, specifically interleukin (IL)-1, 6 and 8, and tumor necrosis factor (TNF)-α, on the ocular surface. These cytokines can disrupt the corneal epithelial barrier, reduce its physical protection and trigger further apoptosis (Massingale et al., 2009; Wu et al., 2020). Thus, the removal of ROS and an increase in antioxidant levels can be an approach for treating DED.

It has been found that therapeutic remedies, such as corticosteroid, anti-inflammatory treatment, and artificial tears, can alleviate DED pathologic symptoms (Barabino et al., 2012; Lin and Yiu, 2014). However, side effects occurred in over 65% of treated patients and relief was not long-lasting (Barabino et al., 2012). Owing to the previously described reasons, a novel therapeutic DED treatment is needed.

For centuries, mushrooms have been an important food source for animals and microorganisms in the ecosystem. A vast number of them also have important pharmacological effects due to their unique constituents (Bhambri et al., 2022). The main constituents of medicinal mushrooms are polysaccharides, phenols, and terpenes, which have antibacterial, antidiabetic, antioxidant, anticancer, anti-inflammatory, and immunomodulatory properties (Elkhateeb et al., 2019).

In this study, six ethanolic extracts were prepared from the medicinal mushrooms *Fomes fomentarius, Ganoderma lucidum, Ganoderma tsugae, Gloeophyllum odoratum, Inonotus oliquus, Laricifomes officinalis* (also known as *Fomitopsis officinalis*). Most of them are widely known for their traditional use (Supplementary Table ST1). The continuously growing body of pharmacological studies highlights the biological effects of fungal extracts, thereby substantiating the traditional medicinal uses of mushrooms: *F. fomentarius* and its bioactive compounds have been reported in the literature to have anti-bacterial (Dresch et al., 2015), anti-cancer (Chen et al., 2008; Kim et al., 2015; Zang et al., 2013), anti-diabetic (Lee, 2005), and anti-inflammatory properties (Park et al., 2004). The use of *G. lucidum* is widespread in Asian countries and it has a long medical history in China for the prevention and treatment of various diseases (Wang et al., 2020). A large number of commercial *G. lucidum* products in various forms, such as powders, dietary supplements and teas made from different parts of the fungus (mycelium, spores and fruiting bodies), are available on the market (Wachtel-Galor et al., 2012). According to the Pharmacopoeia Commission China, the fungus is said to provide relief from nervousness, coughing, insomnia and anorexia (Committee, 2000). It can also be used to regulate blood sugar levels and the immune system, as well as to protect the liver (Wachtel-Galor et al., 2012). *G. tsugae* is closely related to *G. lucidum*. However, while *G. lucidum* grows wild only in Asia and parts of Europe (on broad-leaved trees), *G. tsugae* occurs naturally only in North America on conifers (Luangharn et al., 2021; Zhao et al., 2019). It has been described to possess anti-cancer (Chien et al., 2015; Wang et al., 1993), immunomodulating (Lai et al., 2001; Lin et al., 2006; Won et al., 1992), and wound-healing effects (Su et al., 1999; Yap et al., 2023). The fungus *G. odoratum* has not been widely studied in terms of traditional use and biological-therapeutic potential. However, it has been shown that its thrombin-inhibiting effect is beneficial in preventing blood clotting (Cateni et al., 2015). It has also been demonstrated to neutralise the H3N2 influenza virus (Grienke et al., 2019), scavenge free radicals and suppress phagocytosis (Doskocil et al., 2016). Moreover, *G. odoratum* is rich in fumaric acid (Tel-Çayan, 2019), which is known for its strong antioxidant and antimicrobial properties (Barros et al., 2013; Ribeiro et al., 2008). The sclerotium of *Inonotus obliquus* contain a high level of antioxidants (Lee et al., 2007). The therapeutic effects of the fungus regarding anti-tumour (Mizuno et al., 1999; Taji et al., 2008), immunomodulating (Chen et al., 2019; Kim, 2005), and anti-inflammatory effects (Debnath et al., 2013; Hu et al., 2016; Javed et al., 2019) have been demonstrated *in vitro* and *in vivo*. *L. officinalis* is considered one of the most commonly used medicinal mushroom in European folk medicine (Grienke et al., 2014). Since 2019, *L. officinalis* has been classified as endangered fungal species by the International Union for Conservation of Nature (IUCN) (Kałucka and Svetasheva, 2019). Studies have found a wide range of therapeutic benefits including antiviral (Stamets, 2005), antioxidant (Sha, 2016; Wu et al., 2004), and antimicrobial (Girometta, 2019; Grienke et al., 2014) effects.

## 2 Material and methods

### 2.1 Fungal material and extraction

The fungal materials were either collected and identified by U. Peintner (University of Innsbruck, Austria) or purchased from commercial providers (Supplementary Table ST2). Voucher specimens are deposited at the Division of Pharmacognosy, Department of Pharmaceutical Sciences, University of Vienna, Austria. The taxon names were verified by Mycobank (https://www.mycobank.org/, accessed on 02/04/2024). For each fungal material, approximately 300 mg were extracted twice with 10 mL of 96% (v/v) ethanol in an ultrasonic bath. The obtained extracts were dried using a rotary evaporator at 40°C.

### 2.2 Mycochemcial analysis

UHPLC analysis was conducted on a Waters Acquity UPLC H-Class system equipped with a sample manager, quaternary solvent manager, column manager, PDA detector, ELSD, an isocratic solvent manager, and a quadrupole MS detector (Waters Acquity QDa) with electrospray ionization (ESI) source. For the chromatographic runs, a BEH C_18_ column (1.7 µm, 2.1 × 100 mm, Waters) at 40°C was used. The flow rate was set to 0.3 mL/min with water +0.1% formic acid (A) and acetonitrile + 0.1% formic acid (B) as mobile phases. The following gradient was used: 5% B at 0.0 min, 5%–98% B in 12 min and 98% B for 4 min. Mass detection was performed in positive (cone voltage: 15 V, capillary voltage: 0.8 kV) and negative (cone voltage: 30 V, capillary voltage: 0.8 kV) modes covering a mass range of 100–1,200 Da. A mixture of methanol:water (9:1) + 10 mM ammonium formate was used as a make-up solvent to increase the ionization of investigated metabolites. The instrument was controlled by the software Empower 3.

TLC was performed on Merck silica gel 60 PF254 plates. The mobile phase consisted of dichloromethane:methanol:water (10:1:0.25, v/v/v) and was analyzed at visible light after derivatization with vanillin (1% in methanol)/sulfuric acid (5% in methanol).

### 2.3 HCE-T cell culture

The human corneal epithelial cell-transformed (HCE-T) cell line was obtained from the Riken Cell Bank (RCB2280). Following the provider’s instruction as reported previously (Areesanan et al., 2023). HCE-T cells were cultured in DMEM/F-12 medium (Sigma-Aldrich) supplemented with 5% FCS (BioConcept), 5 μg/mL insulin, 10 ng/mL human epidermal growth factor (EGF), 0.5% DMSO, and 1% penicillin-streptomycin (all from Sigma-Aldrich) at 37°C with 5% CO_2_. The cells were trypsinised with 0.05% trypsin-EDTA solution (Thermo Scientific) at ≥85% confluence. Unless stated otherwise, cells were incubated with the culture medium in a 96-well plate at 2 × 10^4^ cells/well for 24 h to reach over 90% confluence before each experiment. After washing, the medium was substituted with a phenol red-free, non-supplemented, serum-free medium for 24 h to starve the cells before an experiment was conducted.

### 2.4 Free-radical scavenging activity

The free-radical scavenging capacity of the mushroom extracts was tested using a cell-free colorimetric assay based on 2,2 diphenyl-1-picrylhydrazyl (DPPH; Sigma-Aldrich). DPPH changes the colour from violet to pale yellow when the DPPH radical reacts with a hydrogen atom source or electron donor. The study was conducted according to Brand-Williams and colleagues’ protocol with minor modifications (Brand-Williams et al., 1995). To achieve a final concentration of 100 nM of DPPH in a 96-well plate, ethanolic DPPH was thoroughly mixed with a range of concentrations of the mushroom extracts (final concentrations: 1–100 μg/mL). The absorbance was measured spectrophotometrically at 517 nm using a TECAN infinite M Plex after incubation for 30 min at RT in the dark, and the scavenging efficiency was estimated as follows:

$$\mathrm{Free radical scavenging proficiency}\left(\%\right)=\left(\mathrm{blank}-\mathrm{sample}\right)\;/\mathrm{blank}\times 100\%$$

where blank is the DPPH reagent absorbance without any test sample. A rich water-soluble vitamin E derivative, Trolox (100 μM, Tocris Bioscience), was used as a positive control.

### 2.5 Cell viability assay for HCE-T cells

The viability of HCE-T cells treated with the mushroom extracts was determined based on the relative mitochondrial function, as previously reported (Areesanan et al., 2023). The assay is based on the conversion of a tetrazolium salt (WST-1; Sigma-Aldrich) to formazan by cellular mitochondrial dehydrogenases, which is measured by a colour change. Once the HCE-T cells were seeded, grown, and starved in a 96-well plate as mentioned above, the cells were incubated with various concentrations of samples (0.3–100 μg/mL) for 24 h at 37°C with 5% CO_2_. Afterwards, the cells were washed with phosphate-buffered saline (PBS; Sigma-Aldrich), incubated with WST-1 (1:10) for 2 h at 37°C and then analyzed spectrophotometrically at 450 nm, with 650 nm as reference using a TECAN infinite M Plex. Triton X-100 (5% v/v, Sigma-Aldrich), a detergent used to lyse cells, was used as a positive control.

### 2.6 HCE-T intracellular ROS measurement

The intracellular ROS levels of UVB-exposed HCE-T cells were determined by the emitted signal of 2′,7′-dichlorodihydrofluorescein diacetate (H_2_DCFDA; Invitrogen) using the method, as previously described (Areesanan et al., 2023). Briefly, HCE-T cells were incubated with 25 μM H_2_DCFDA probe for 30 min at 37°C, washed, and treated with the mushroom extracts (1–30 μg/mL) for 10 min at 37°C. Subsequently, the treated cells were exposed to UVB radiation at 312 nm, an intensity of 5.45 mW/cm^2^, and a dose of 50 mJ/cm^2^ (Vilber Lourmat) and incubated for a further 30 min at 37°C. The fluorescence intensity (488/530 nm) was measured spectrophotometrically using a TECAN infinite M Plex. Trolox (100 μM, Tocris), a potent antioxidant, was used as a positive control.

### 2.7 HCE-T wound healing assay

In this study, HCE-T cells were scratched and treated with the fungal extracts to determine the efficacy of wound healing, according to the reported method (Areesanan et al., 2023). HCE-T cells (2 × 10^4^ cells/well of 96-well plate) for 24 h in the culture medium until confluence was reached. The medium was then replaced for 24 h with a supplement-reduced medium (DMEM/F-12 medium supplemented with 1% penicillin-streptomycin and 5% FCS) to delay the cell migration. Using AutoScratch (BioTek), consistently cell-free (wound) gaps were made across each well. The wells were washed twice to remove detached cells before treating the cells overnight with the supplemented-reduced medium mixed with different concentrations of the samples (3–30 μg/mL). The images of the gap areas were taken at 17 h post-scratch using an inverted microscope (Zeiss) and the gap closure was quantified using I Fiji ver. 2.14.0/1.54f (Schindelin et al., 2012) with an open-source *Wound Healing Size Tool* plugin (Suarez-Arnedo et al., 2020). An alkyl hydroperoxide, *tert*-Butyl hydroperoxide (25 μM, Sigma-Aldrich), was used as a control that impedes wound healing.

### 2.8 HCE-T TEER and fluorescein permeability assay

HCE-T cells (3 × 10^4^ cells) were inoculated with 400 μL of culture medium onto a translucent polyester (PET) membrane transwell insert (0.4 μm pore size; Greiner bio-one), and 1 mL of culture medium was added into the basolateral chamber. The cells were kept for 96 h at 37°C. Following washing twice, the cells were treated with the samples cultured in a phenol red-free non-supplemented serum-free medium. Over the course of 72 h, transepithelial electrical resistance (TEER, CellZscope) was measured every 24 h. Afterwards, the cells were washed twice with Hank’s Balanced Salt solution (HBSS; Gibco), before 50 μM of sodium fluorescein (Sigma-Aldrich) was added into the inserts and 1 mL of HBSS was added into the basolateral chamber. After incubating the cells for 2 h at 37°C, 200 μL of the basolateral side’s media was taken and spectrometrically quantified at 490 nm (TECAN unlimited M Plex).

### 2.9 HCE-T secreted cytokine quantification

After a 24-h fasting period in a 96-well plate containing 2 × 10^4^ cells/well, the HCE-T cells were treated with samples (3–30 μg/mL) for 10 min at 37°C. The cells were then subjected to a UVB lamp (312 nm, 5.45 mW/cm2, 50 mJ/cm2, Vilber Lourmat) and were incubated for an additional 24 h at 37°C. The supernatants were collected and stored at −80°C. To quantify the secreted mediators (IL-6, TNF-α, and IL-8), the experiment was conducted following the manufacturer’s instructions for the multiplex bead-based assay kit (LEGENDplex™, BioLegend), which based on the principle of capture bead-analyte-detection antibody sandwiches bound with streptavidin-phycoerythrin (SA-PE). The bead captures signified the types of the mediators and the fluorescent intensity was measured through SA-PE. The analysis was conducted using a flow cytometer (Beckman Coulter) and FlowJo software. Dexamethasone (10 μM, Sigma-Aldrich) was used as a positive control to inhibit cytokine secretion.

### 2.10 THP-1 NFκB-eGFP cell culture

Human monocytic THP-1 NFκB-eGFP reporter cells (Merck) were used to depict the mushroom extracts’ effects on immune cells. The cell line was cultured in RPMI-1640 medium supplemented with 2 mM L-glutamine (Sigma-Aldrich), 10% heat-inactivated FCS (BioConcept), and 1% penicillin-streptomycin (Sigma-Aldrich). The cells were maintained at 37°C with 5% CO_2_ atmosphere.

### 2.11 Cell viability assay for THP-1 cells

WST-1 assay was used to determine the impact of the mushroom extracts on the viability of the THP-1 cells. In a 96-well plate, THP-1 (5 × 10^4^ cells/well) were seeded together with the extracts (0.3–100 μg/mL) in a serum-free media for 24 h at 37°C. The treated cells were centrifuged at 300 g for 5 min to remove the supernatants, washed twice, and the cells were further incubated with 1:10 WST-1 for 2 h at 37°C. The absorbance was then measured (450 nm/650 nm as a reference; TECAN infinite M Plex). Triton-X-100 (1% v/v, Sigma-Aldrich) was used as a positive control.

### 2.12 THP-1 secreted cytokine quantification

In serum-free media together with lipopolysaccharide (LPS, 1 μg/mL), THP-1 cells (5 × 10^4^ cells/well of 96-well plate) were incubated with the samples (3–30 μg/mL) for 48 h at 37°C. The supernatants were collected and stored at −80°C until further analysis. Detection of supernatant’s mediators (IP10, IL-8, TNF-α, and IL-6) was achieved through a multiplex bead-based flow cytometry assay (LEGENDplex, BioLegend) similar to the analysis of the HCE-T supernatants. Dexamethasone (DEX, 10 μM, Sigma-Aldrich) was used as a positive control.

### 2.13 IHMGECs cell culture

Immortalized human meibomian gland epithelial cells were incubated at 37°C with 5% CO_2_ in culture medium (Keratinocyte Serum Free Serum (FM) (1X), EGF Human Recombinant, Bovine Pituitary Extract; all from Thermo Fisher Scientific). Before each experiment, the cells were induced to a mature stage. To induce differentiation, cells were grown in a serum-containing medium which consisted of DMEM/F-12 medium (Sigma- Aldrich), supplemented with 4% FCS (BioConcept), and 10 ng/mL human EGF (Sigma-Aldrich) for 24 h at 37°C with 5% CO_2_.

### 2.14 Cell viability assay for IHMGECs

The cytotoxicity of the samples was evaluated using the WST-1 colorimetric assay. IHMGECs (ATCC; 2 × 10^4^cells/well) were seeded in a 96-well plate and incubated at 37°C with 5% CO_2_ for 72 h in the culture media. After the cells were washed, the cells were incubated with the serum-containing medium mixed with the samples (0.3–100 μg/mL) for 24 h followed by 2 h with WST-1 (Sigma-Aldrich) at 37°C. The metabolic activity was quantified *via* scanning at 450 nm with 650 nm as a reference for the spectrometric analysis. Triton-X-100 (5% v/v, Sigma-Aldrich) was used as a positive control.

### 2.15 IHMGECs intracellular ROS measurement

IHMGECs (2 × 10^4^cells/well) were cultured in a 96-well plate for 72 h in the culture medium. Afterwards, the cells were washed and incubated in the 4% differentiation medium for 24 h at 37°C. The cells were washed at incubated with 10 μM H_2_DCFDA (Invitrogen) for 30 min at 37°C in the 0% FCS differentiation medium, before treating with or without the fungal extracts for 10 min at 37°C. The cells were then irradiated with the UVB (312 nm, 5.45 mW/cm^2^, 50 mJ/cm^2^; Vilber Lourmat). After further incubation for 30 min, the emitted signal was spectrophotometrically measured (488/530 nm; TECAN infinite M Plex). Trolox (100 μM, Tocris) was used as a positive control.

### 2.16 Lipid staining of meibomian gland cells

With a few minor modifications, LipidTOX (Invitrogen) was utilised to monitor the effects of the samples on the cells and lipid synthesis, following the description of Liu and colleagues (Liu et al., 2014) with modifications. IHMGECs (3.5 × 10^4^ cells/well) were incubated on a sterile glass coverslip in each well of a 24-well plate for 72 h at 37°C with the growth medium. The cells were treated for 24 h (3 and 30 μg/mL or 1 and 10 μg/mL) in the 4% FCS differentiation medium after being washed with PBS. The control groups were incubated in a differentiation medium containing 10% FCS to strongly stimulate lipid synthesis. After a 24 h period, the cells were rinsed and fixed with cold 4% paraformaldehyde (PFA, Electron Microscopy Sciences) for 15 min at room temperature, and then counter-stained for 30 min at RT in the dark using a mixture of LipidTox (1:500, Invitrogen) and DAPI (400 nM, Invitrogen). Once the cells had been thoroughly rinsed, the coverslips were taken off the wells and ProlongTM Diamond Antifade Mountant (Thermo Fisher Scientific) was used to mount the cells on a glass slide. The cells were then kept in storage at 4°C. Each sample was subjected to a total of 5 random photographs using a widefield fluorescence microscope (Nikon Ti2).

Using Fiji (ver. 2.14.0/1.54f), the lipid droplets:cells ratio and their size were measured to evaluate the lipid synthesis of IHMGECs (Schindelin et al., 2012). In summary, each sample’s Z-position pictures were combined into a single image using the maximum intensity pixels criterion and stored in the 16-bit.tif format at 5.45 DPI. Rolling ball background subtraction algorithms were applied with a radius of 8 pixels for the droplets and a radius of 4 pixels for the background to create images of reduced background noise and a blurred background, respectively, in order to adjust the uneven background intensity and remove areas with spatial intensity variations. To clearly distinguish between the two, the decreased background noise image was subtracted from the blurred background image. The reduced background noises image was subtracted from the blurred background image to provide an intensity value of 0 for the droplets, which allowed them to be easily distinguished from the background. After that, the invert function was used to give the droplets more attention, resulting in all of the droplets having larger values than the others. This was followed by the implementation of a second rolling ball function with 4-pixel radius. Gaussian blur function then was used to remove any dust-effect particles followed by another rolling ball function with 8-pixel radius. To generate a mask, the auto-threshold function default was used. Then, with three erosion cycles, the clumped lipid droplets were separated using the Watershed Irregular Features algorithm. Ultimately, the analyze Particles function was used to count and analyze each droplet. Similarly, to count the number of cells in each sample’s picture and to separate clumped DAPI-stained cells, a mask was made using the Watershed function.

### 2.17 Statistical analysis

Statistical data analysis was performed using PRISM version 10.3.1 (GraphPad software). Every experiment was carried out separately at least three times, and the values that are given are the means ± standard deviation. One-way ANOVA was used to assess statistical significance, along with Dunnett’s test and the Brown-Forsythe test to ensure homogeneity of variance. Statistical significance was assumed at *p < 0.05, **p < 0.01, ***p < 0.001, ****p < 0.0001.

### 2.18 Declaration of generative AI and AI-assisted technologies in the writing process

During the preparation of this work the authors used DeepL Translator in order to improve language. After using this tool/service, the authors reviewed and edited the content as needed and take full responsibility for the content of the publication.

## 3 Results

### 3.1 Mycochemical profiling of fungal extracts

**FIGURE 1:**
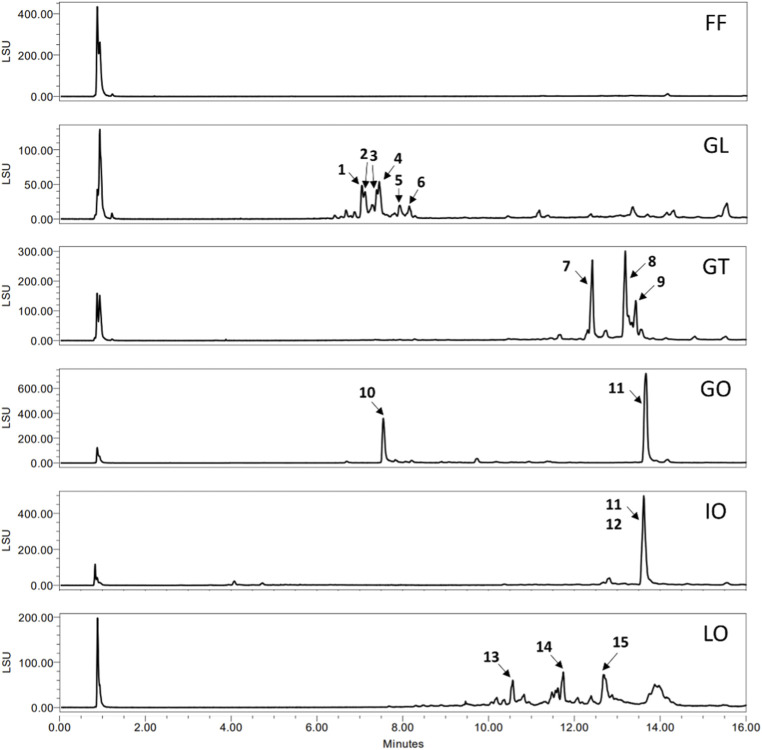
UHPLC-ELSD chromatograms of fungal extracts containing annotated constituents.

The six fungal extracts of the fruit bodies of *F. fomentarius* (FF)*, G. lucidum* (GL)*, G. tsugae* (GT)*, G. odoratum* (GO)*, L. officinalis* (LO), and the sclerotium of *I. obliquus* (IO) were analyzed by UHPLC-ELSD/-MS and TLC to gain insights into their mycochemical composition (Figure 1; Supplementary Figure SF1). The main constituents found in the ELSD chromatograms were characterized based on the recorded MS data and literature (Table 1). In extract GL, highly oxygenated lanostane triterpenes (**1**–**6**) were tentatively annotated as the main constituents which are well-known secondary metabolites of *G. lucidum* fruit body in literature (Wu et al., 2017). In contrast, extracts GT, GO, IO and LO consist of more lipophilic constituents which is reflected by the later RTs of these compounds. For extract GT, three compounds were tentatively annotated as acetoxylated triterpenes (**7**–**9**) and their potential molecular formulas were proposed. One triterpene was found in the extracts GO and IO and was annotated as trametenolic acid B (**11**) (Grienke et al., 2019). A second compound co-eluting with the peak of trametenolic acid B was dereplicated as inotodiol (**12**) in the extract IO. Both triterpenes are known to be the major compounds in *I. obliquus* fruit body (Kim et al., 2020). In addition, another triterpene (**10**) was tentatively annotated in extract GO for which a molecular formula was established. Considering its rather short retention on the column, it might be a more hydrophilic triterpene but could not be dereplicated by literature. In extract LO, malonylated triterpenes (**13**–**15**) were annotated based on MS data and literature (Han et al., 2016). No triterpenes were detected in extract FF which only showed a large peak in the beginning of the ELSD chromatogram as it is the case for GL and LO. Within that peak, *m/z* values of 365.1 [M + Na]^+^ in positive mode and 341.1 [M-H]^-^ in negative mode were detected and assigned to a disaccharide which might be derived from polysaccharides upon MS ionisation. Besides low molecular weight secondary metabolites such as triterpenes, polysaccharides are among the most investigated chemical compounds of polypores with various reported biological properties (Grienke et al., 2014). In addition, the TLC fingerprint analysis revealed purple spots after spraying with vanillin/sulfuric acid in each extract, suggesting the presence of terpenoids at least as minor constituents in all extracts.

**TABLE 1:** Tentative MS-based annotation of constituents detected in the investigated fungal extracts (as labelled in Figure 1).

No.	RT [min]	Positive mode [*m/z*]	Negative mode [*m/*z]	Molecular formula	Putative annotation
1	7.04	497.3 [M + H-2x H_2_O]^+^, 515.3 [M + H-H_2_O]^+^, 555.3 [M + Na]^+^	495.4 [M-H-2x H_2_O]^-^, 513.4 [M-H-H_2_O]^-^	C_30_H_44_O_8_	ganoderic acid G
2	7.14	499.3 [M + H-H_2_O]^+^, 517.2 [M + H]^+^, 539.3 [M + Na]^+^	497.4 [M-H-H_2_O]^-^, 515.3 [M-H]^-^	C_30_H_44_O_7_	ganoderic acid A or B
3	7.38	573.3 [M + H]^+^, 595.3 [M + Na]^+^	553.4 [M-H-H_2_O]^-^	C_30_H_44_O_9_	ganoderic acid H
4	7.44	499.3 [M + H-H_2_O]^+^, 539.3 [M + Na]^+^	515.4 [M-H]^-^	C_30_H_44_O_7_	ganoderic acid A or B
5	7.95	497.3 [M + H-H_2_O]^+^, 515.3 [M + H]^+^, 537.3 [M + Na]^+^	495.3 [M-H-H_2_O]^-^	C_30_H_42_O_7_	ganoderic acid C or D
6	8.14	571.3 [M + H]^+^, 593.3 [M + Na]^+^	551.38 [M-H-H_2_O]^-^	C_32_H_42_O_9_	ganoderic acid F
7	12.42	493.4, 535.3 [M + H- CH_3_COOH-H_2_O]^+^, 630.5 [M + NH_4_]^+^ 635.4 [M + Na]^+^	-	C_36_H_52_O_8_	threefold acetoxylated triterpene
8	13.12	435.4 [M + H-2x CH_3_COOH]^+^, 495.5	-	C_34_H_50_O_6_	twofold acetoxylated triterpenes
9	13.43	[M + H-CH_3_COOH]^+^, 577.4 [M + Na]^+^			
10	7.54	467.4, 485.3, 525.3 [M + Na]^+^	501.4 [M-H]^-^	C_30_H_46_O_6_	highly oxygenated triterpene
11	13.65	439.4 [M + H-H_2_O]^+^, 457.3 [M + H]^+^	-	C_30_H_48_O_3_	trametenolic acid B
12	13.65	425.4 [M + H-H_2_O]^+^	-	C_30_H_50_O_2_	inotodiol
13	10.60	571.4 [M + H-H_2_O]^+^, 611.4 [M + Na]^+^	543.5, 587.5 [M-H]^-^, 609.5	C_35_H_56_O_7_	malonylated triterpene
14	11.75	585.4 [M + H-H_2_O]^+^, 625.4 [M + Na]^+^	601.5 [M-H]^-^	C_35_H_53_O_8_	officimalonic acid E
15	12.73	567.4 [M + H-H_2_O]^+^, 607.4 [M + Na]^+^	-	C_34_H_48_O_8_	officimalonic acid B

### 3.2 Toxicity, antioxidant properties and wound healing capacity of the mushroom extracts

**FIGURE 2:**
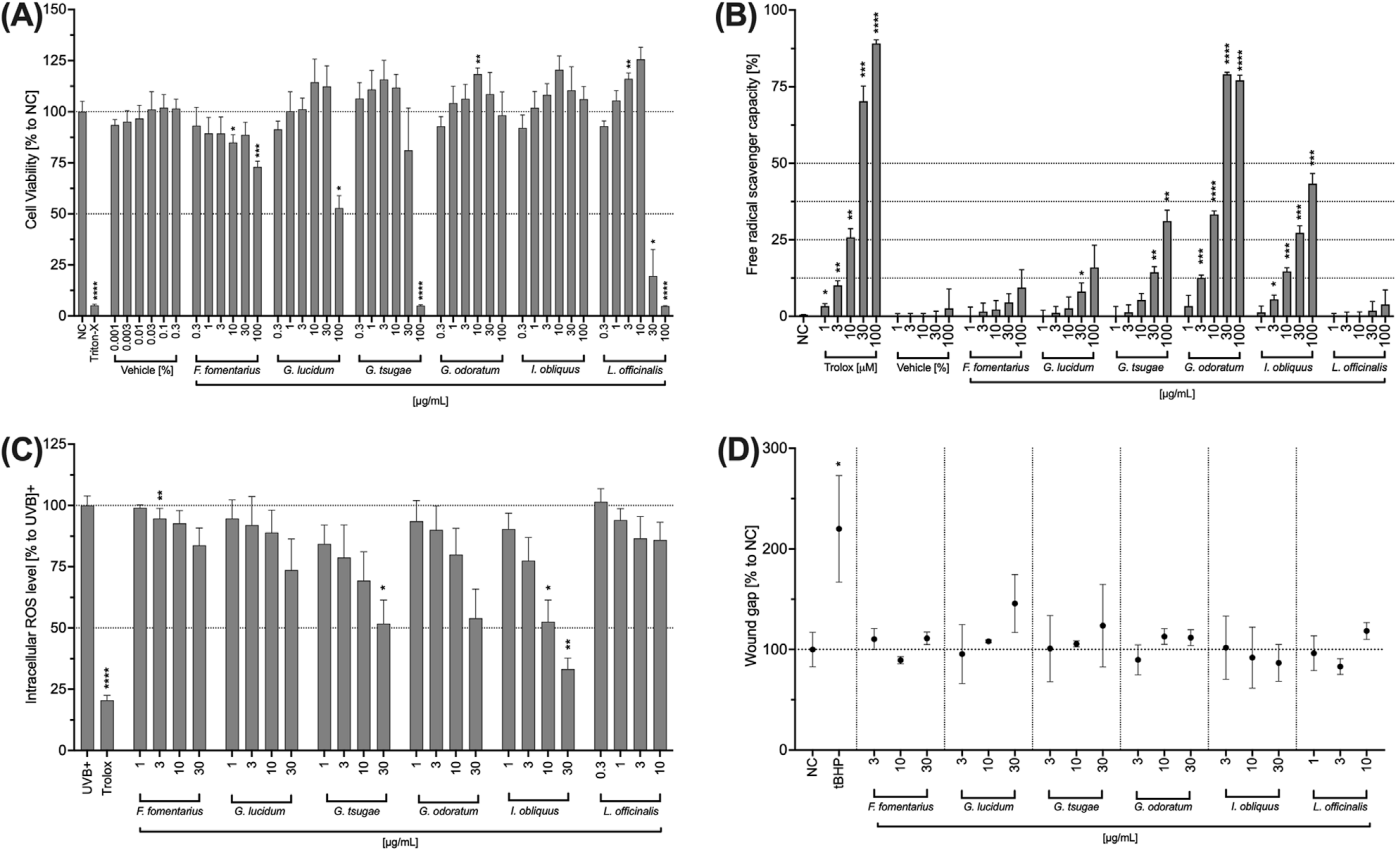
Effects of fungal extracts on the viability **(A)**, radical scavenging capacity **(B)**, intracellular ROS level **(C)**, and wound healing **(D)** of HCE-T cells. **(A)** HCE-T cells were incubated for 24 h with medium (NC), Triton X-100 (Triton-X; 1% v/v), DMSO (vehicle) or the fungal extracts. The number of viable cells was determined and normalised to the untreated control and expressed as mean ± standard deviation. **(B)** In a cell-free assay, DMSO (vehicle) and the mushroom extracts were incubated with DPPH (100 µM) for 30 min and the free radical scavenging capability was quantified. Trolox was used as positive control. The mean fluorescence intensity was determined and plotted ±standard deviation. **(C)** HCE-T cells were treated with H_2_DCFDA (25 µM) for 30 min and incubated with the fungal extracts for 10 min before exposure to a UV lamp. The fluorescence intensity was measured and the values were normalised to the control (UVB) and presented as mean ± standard deviation. **(D)** HCE-T cells were scratched and incubated overnight with different concentrations of mushroom extracts. Images were taken with a digital camera and analysed. The wound size was normalised to the control (untreated) and expressed as mean ± standard deviation. All experiments were independently repeated (n = 3) and *p < 0.05, **p < 0.01, ***p < 0.001, ****p < 0.0001.

In the first step, cytotoxic concentrations of the fungal extracts were determined. For this purpose, a viability test was performed after 24 h of incubation of HCE-T cells with the fungal extracts. Triton-X 100 (1% v/v) was used as a positive control that significantly reduced the viability of the HCE-T cells. FF, GL, and GT significantly reduced the viability of HCE-T cells at a concentration of 100 μg/mL (Figure 2A). Among them, GT showed the highest toxicity with cell viability comparable to Triton-X 100 (Figure 2A). LO is highly toxic to HCE-T cells as at concentrations of 30–100 μg/mL, the viability of HCE-T cells was significantly below 50% (Figure 2A). Therefore, the concentration range of LO was adjusted hereinafter and lower concentrations were used for the functional tests. GT and IO showed no toxicity over the entire concentration range (0.3–100 μg/mL) (Figure 2A).

Exposure to UVB can lead to an accumulation of ROS and subsequently to corneal damage and inflammation. Therefore, the ability of the mushroom extracts to reduce ROS as well as their radical scavenging capacity was investigated. All mushroom extracts exhibited free radical scavenging capacity in a concentration-dependent manner (Figure 2B). However, for the extracts FF, GL and LL, this increase was minor and without significant effects. For GT, a significant free radical scavenging capacity was observed at concentrations of 30 and 100 μg/mL while for GO and IO, the free radical scavenging capacity was significant in the concentration range of 3–100 μg/mL.

All mushroom extracts decreased the generation of ROS in HCE-T cells concentration-dependently compared to the untreated control (Figure 2C). In a dose-dependent manner, the strongest ROS reduction was observed in IO followed by GT, GO, and GL. Significantly lower ROS levels were obtained for 30 μg/mL of GT and 30 and 10 μg/mL of IO (Figure 2C).

Since corneal damage is one of the symptoms of DED, the influence of the extracts on the wound healing capacity of HCE-T cells was also investigated below. None of the extracts significantly promoted wound healing compared to the untreated control (NC) (Figure 2D). However, slight concentration-dependent positive effects on wound healing were observed for GL and GT (Figure 2D). Nevertheless, these effects did not become significant.

### 3.3 Effects of the fungal extracts on the TEER of HCE-T cells

**FIGURE 3:**
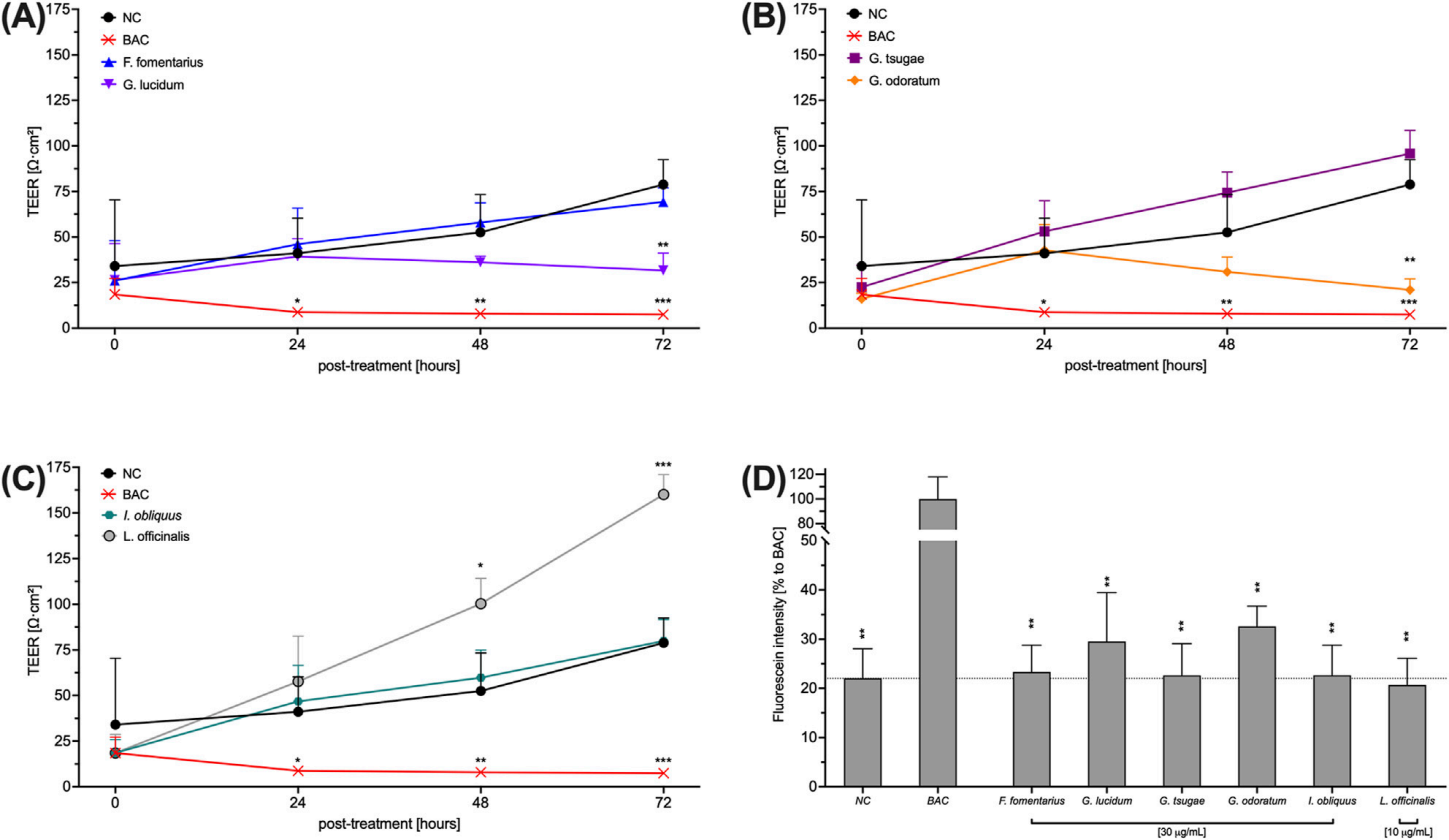
Effects of fungal extracts on the TEER **(A, B, C)** and fluorescein permeability **(D)** of HCE-T cells. **(A, B, C)** HCE-T cells were cultured on a translucent membrane of a transwell insert for 72 h and treated with mushroom extracts. The TEER was measured every 24 h. **(D)** After TEER measurement, cells were washed and exposed to fluorescein for 2 h to determine permeability. BAC was used as a positive control. Data were normalised to 4% FCS and presented as mean ± standard deviation. n = 3; *p < 0.05, **p < 0.01, ***p < 0.001.

One of the symptoms of DED is the appearance of corneal membrane damage. To evaluate the use of mushroom extracts in DED, their effect on the tight junctional connection of HCE-T cells was measured by TEER. Therefore, cells were incubated with the extracts for 72 h and the TEER was measured every 24 h. Benzalkonium chloride (BAC) disrupts tight junctions and was therefore used as a control. The influence of the fungal extracts on the TEER of HCE-T cells is somewhat divergent. While GL (Figure 3A) and GO (Figure 3B) slightly decreased the TEER compared to the untreated control (NC), treatment with LO (Figure 3C) led to a significantly increased TEER. The TEER after treatment with FF (Figure 3A), GT (Figure 3B) and IO (Figure 3C) was comparable to the untreated control (NC). The fluorescein permeability of HCE-T cells (Figure 3D) was largely comparable to that of the untreated control (NC). Only GL and GO showed slightly increased values.

### 3.4 Effects of mushroom extracts on the cytokine secretion of HCE-T cells

In the following, the influence of the mushroom extracts on the secretion of the pro-inflammatory cytokines IL-6, TNF-α and IL-8 by HCE-T cells was investigated. In addition to its inflammatory effect, IL-6 accelerates wound healing of HCE-T cells and TNF-α also promotes re-epithelialisation and cell proliferation depending on IL-6 and IL-8. The HCE-T cells were exposed to UVB and treated with the fungal extracts for 24 h. Subsequently, IL-6, TNF-α and IL-8 levels were determined by Legendplex™ assay.

**FIGURE 4:**
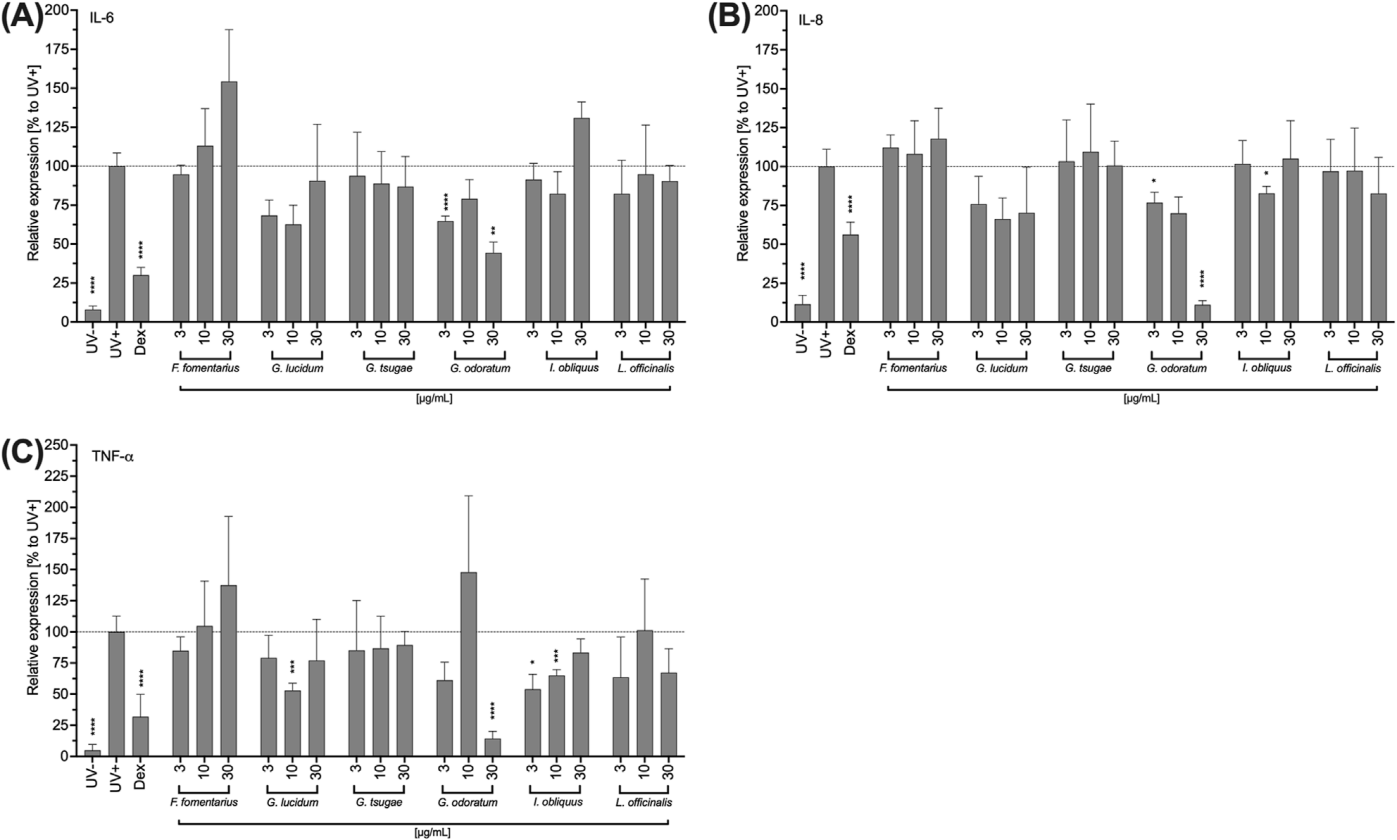
Impact of fungal extracts on the IL-6 **(A)**, IL-8 **(B)** and TNF-a **(C)** cytokine secretion of HCE-T cells. HCE T cells were exposed to UV light for 24 h and treated with DMSO (vehicle) or the fungal extracts. After incubation with the extracts, the supernatants were collected to quantify the secreted mediators using LEGENDplex™. As an inhibition control, the cells were treated with 10 µM dexamethasone (DEX) prior to UVB exposure. Relative cytokine secretions were normalised to the UVB-stimulated control (Stim.) and presented as mean ± standard deviation. n = 3; *p < 0.05, **p < 0.01, ***p < 0.001, ****p < 0.0001.

In contrast to other extracts, FF-treated HCE-T increased the pro-inflammatory cytokines levels in a concentration-dependent but not significant manner (Figure 4A). IO increased IL-6 secretion only at the highest concentration (30 μg/mL) tested, while GL and GO inhibited the IL-6 secretion of HCE-T cells (Figure 4A). For GO, this inhibition was significant at concentrations of 3 and 30 μg/mL (Figure 4A). Moreover, inhibition of IL-8 secretion was observed after treatment with GL and GO (Figure 4B). For GO, this inhibition was concentration-dependent with significances at concentrations of 3 and 30 μg/mL (Figure 4B). In addition, FF increased TNF-α secretion in a concentration-dependent manner, but not significantly (Figure 4C), whereas GL, GO, and IO inhibited the secretion of TNF-α (Figure 4C). Significant effects were observed for 10 μg/mL of GL, 30 μg/mL of GO, and 3 and 10 μg/mL of IO (Figure 4C).

### 3.5 Effects of mushroom extracts on the cytokine secretion of THP-1 cells

**FIGURE 5:**
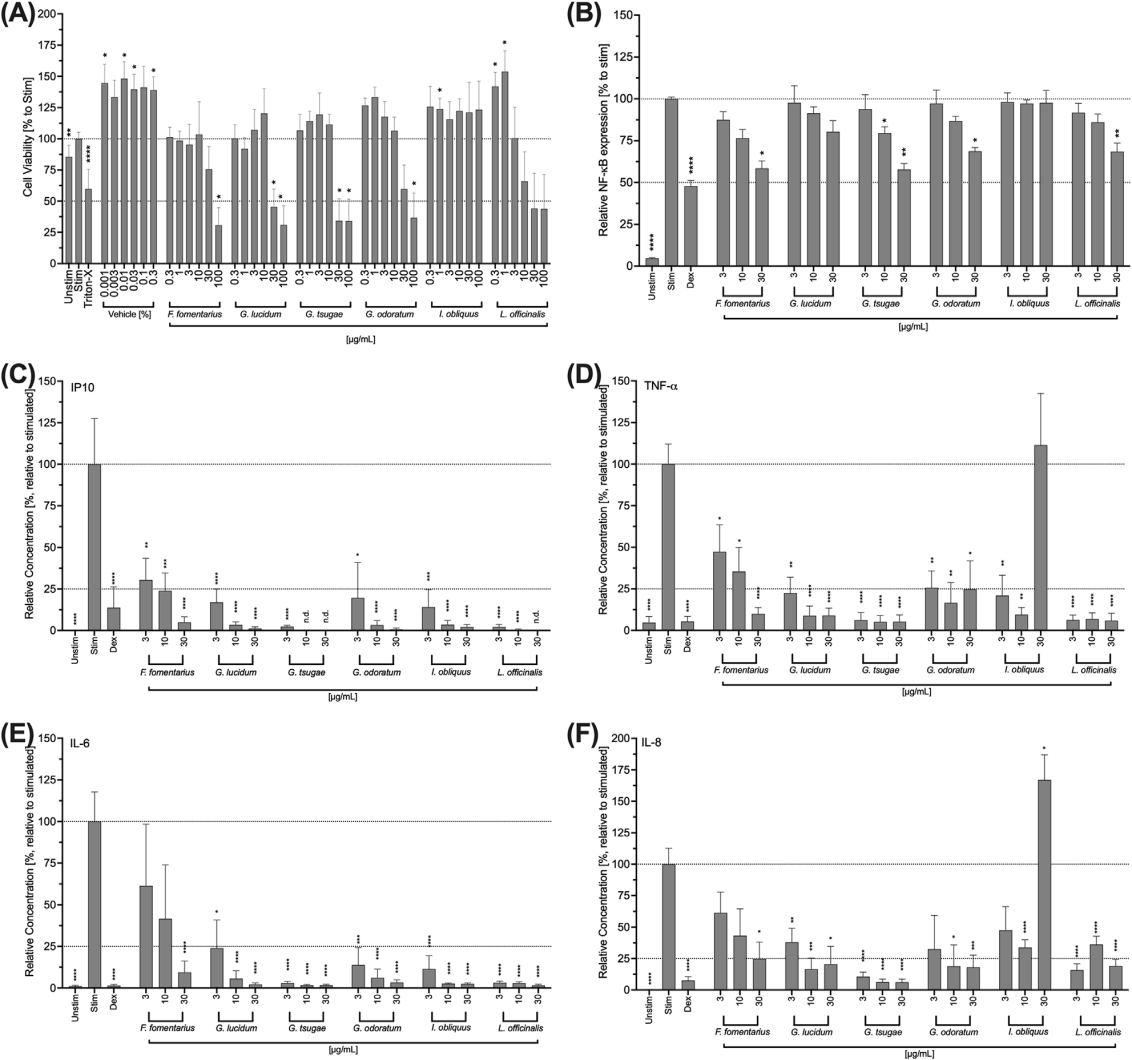
Impact of fungal extracts on the viability **(A)**, NF-κB expression **(B)**, IP10 **(C)**, TNF-a **(D)**, IL-6 **(E)**, and IL-8 **(F)** cytokine secretion of THP-1 cells. **(A)** THP-1 cells were treated with DMSO (vehicle) or the fungal extracts for 24 h. Their viability was determined using the colorimetric WST-1 assay. Triton-X 100 (Triton-X; 1% v/v) was used as a control. The data were normalised to the negative control (Unstim) and expressed as mean ± standard deviation. n = 3; *p < 0.05, **p < 0.01, ****p < 0.0001. **(B)** Retroviral transduced NF-κB-eGFP-THP-1 human monocytic cells were stimulated with LPS (100 ng/mL) and treated with the mushroom extracts for 24 h. Dexamethasone (Dex) was used as an inhibitor control. NF-κB expression was analysed by flow cytometry. Relative NF-κB expression was normalised to the control (Stim) and presented as the mean ± standard deviation. n = 3. *p < 0.05, **p < 0.01, ****p < 0.0001. **(C, D, E, F)** THP-1 cells were stimulated with LPS (100 ng/mL) and treated with the fungal extracts for 48 h. Dexamethasone (Dex) was used as an inhibitor control. The supernatants were collected and analysed using LEGENDplexTM. Relative cytokine concentration was normalised to the control (Stim.) and presented as mean ± standard deviation. n = 3; *p < 0.05, **p < 0.01, ***p < 0.001, ****p < 0.0001.

Since the clinical picture of DED is characterized by inflammation of the cornea, the influence of the fungal extracts on the THP-1 immune cell line was investigated below. First, cytotoxic concentrations were determined analogously to the HCE-T cells. For GL and GT, the concentrations 30 μg/mL and 100 μg/mL proved to be significantly cytotoxic for the THP-1 cells (Figure 5A). Significant cytotoxic effects also occurred for FF and GO at a concentration of 100 μg/mL (Figure 5A). In THP-1 cells, LO showed a concentration-dependent reduction of cell viability between 10 and 100 μg/mL (Figure 5A). However, none of the tested concentrations was significantly cytotoxic (Figure 5A). IO was well-tolerated in the entire concentration range (Figure 5A).

NF-κB is important for the signal transduction required for the inflammatory function of THP-1 cells. For this reason, the influence of the fungal extracts on the expression of NF- κB in THP-1 cells was evaluated. Except for IO, all extracts inhibited NF-κB expression in a concentration-dependent manner (Figure 5B). For FF, GO and LO, this inhibition was significant at a concentration of 30 μg/mL (Figure 5B). A significant inhibition of NF-κB expression was observed for GT at concentrations of 10 and 30 μg/mL (Figure 5B). However, at a concentration of 30 μg/mL, this extract also had significant cytotoxic effects on THP-1 cells (Figure 5B).

In order to analyze a possible effect of the mushroom extracts in inflammation of the cornea associated with DED, the cytokine secretion of THP-1 cells was also investigated in analogy to the HCE-T cells. For this purpose, the cells were treated with the extracts for 24 h, the supernatants were collected and the IP-10, IL-6, TNF-α, and IL-8 levels were determined. All mushroom extracts strongly and significantly inhibited the secretion of all cytokines in a concentration-dependent manner with significantly reduced levels for the entire concentration range (3–30 μg/mL) (Figures 5C–F). However, treatment of the cells with 30 μg/mL IO extract increased TNF-α (Figure 5D) and IL-8 (Figure 5F) levels, while 3 and 10 μg/mL of IO extract lowered TNF-α (Figure 5D) and IL-8 (Figure 5F) secretions. FF showed the weakest inhibitory effects at the lowest concentration tested (3 μg/mL). This was most noticeable in the results of the IL-6 measurement.

### 3.6 Effects of mushroom extracts on the viability and lipid droplets of immortalized human meibomian gland epithelial cells (IHMGECs)

DED is characterized, in part, by a deficient quality of the tear film produced by the meibomian gland cells. Therefore, the effects of mushroom extracts on IHMGECs viability, their intracellular ROS level, lipid-to-cell ratio, lipid size, cell number and cell size were investigated in the following. To differentiate the IHMGECs into a highly lipid-producing form, the cells were incubated with a differentiation medium containing 10% inactivated fetal calf serum (iFCS). To stimulate lipid production to a lesser extent or not at all, a differentiation medium with 4% iFCS or no iFCS was used. Cells incubated in the culture medium (CM) were used to represent the undifferentiated form.

**FIGURE 6:**
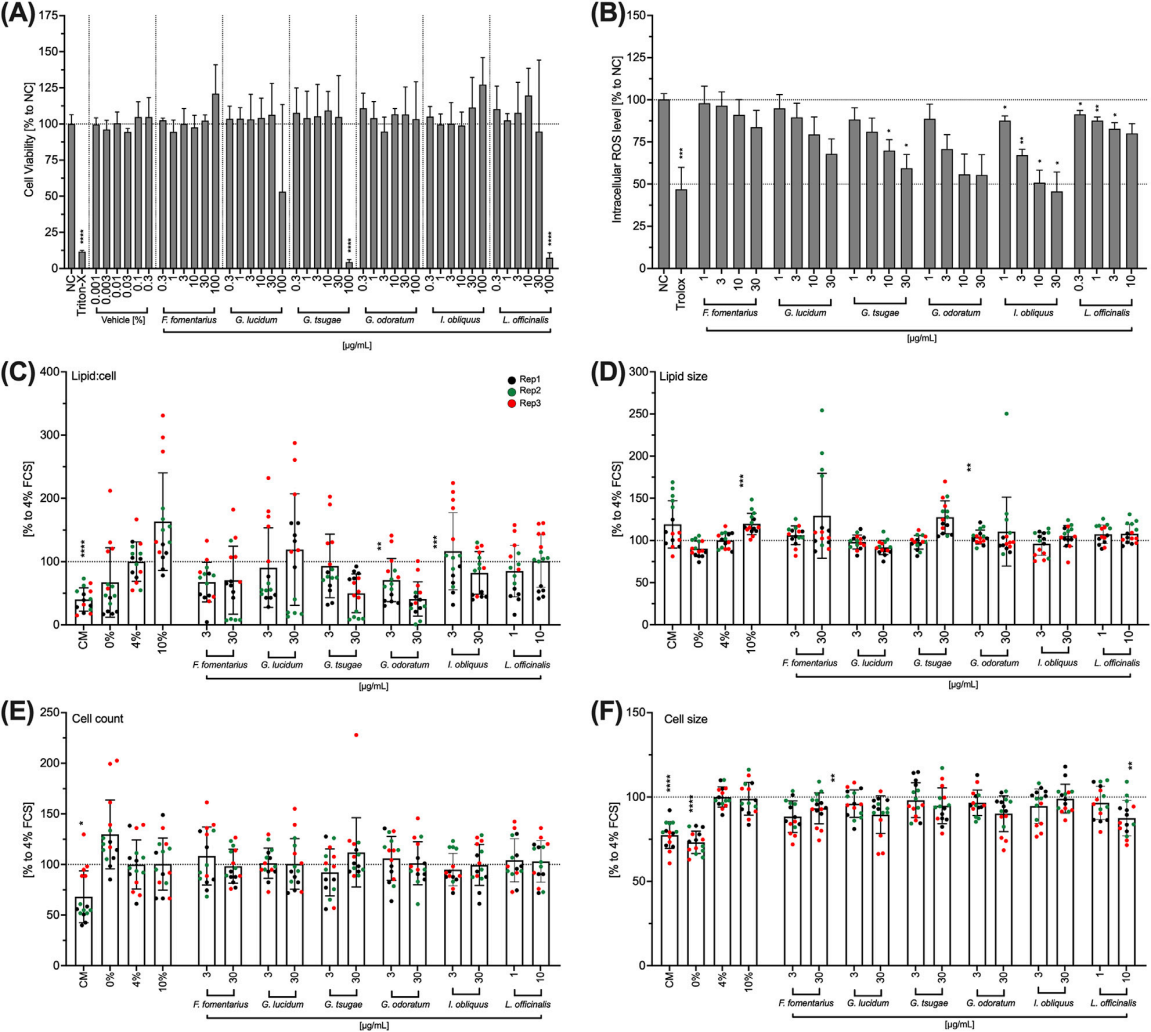
Effects of mushroom extracts on IHMGEC viability **(A)**, intracellular ROS level **(B)**, lipid droplet/cell ratio **(C)**, lipid droplet size **(D)**, cell number **(E)** and cell size **(F)**. **(A)** Confluent IHMGECs were incubated with DMSO (vehicle) or the mushroom extracts in 4% differentiation medium for 24 h before cell viability was measured using WST-1. Triton-X 100 (Triton-X; 1% v/v) was used as a control. **(B)** IHMGECs were treated with H_2_DCFDA (10 µM) for 30 min and incubated with the fungal extracts for 10 min before exposure to a UV lamp. The fluorescence intensity was measured and the values normalised to the control (NC) and presented as mean ± standard deviation. n = 3; *p < 0.05, **p < 0.01, ***p < 0.001. **(C,D,E, F)** IHMGECs were incubated for 24 min 4% FCS differentiation medium with or without fungal extracts. Cells incubated in CM, 0% FCS or 10% FCS were used as controls. A cocktail of LipidTOX and DAPI was used to evaluate the lipid droplets using a fluorescence microscope and Fiji software. Data were normalised to 4% FCS and presented as mean ± standard deviation. Replication = Rep; n = 3; **p < 0.01, ***p < 0.001, ****p < 0.0001.

GT and LO significantly reduced cell viability at a concentration of 100 μg/mL (Figure 6A). The lower concentrations tested had no effect on cell viability (Figure 6A). 100 μg/mL of GL also reduced the viability of iHMGECs; however, this inhibition was not significant due to a very high standard deviation (Figure 6A). All other extracts had no effect on the viability of IHMGECs in the entire concentration range (0.3–100 μg/mL) (Figure 6A). The ROS level was reduced in a concentration-dependent manner by all fungal extracts (Figure 6B), particularly for FF, GT, GO, and GL. This inhibition was significant for GT at concentrations of 10 and 30 μg/mL, for IO in the entire concentration range (1–30 μg/mL) and for LO in the concentration range of 0.three to three μg/mL (Figure 6B). Nevertheless, the ROS effect of LO was observed in the toxic concentrations. The lipid-to-cell ratio, cell number and cell size of IHMGECs were not affected by any of the extracts in the tested concentrations (Figures 6C–F).

## 4 Discussion

DED is a complex, prevalent ocular surface disease, and its aetiology is still poorly understood. However, significant oxidative stress and inflammatory processes appear to play an important role (Wei and Asbell, 2014). Medicinal mushrooms embody a valuable ethnopharmacological source and therefore the pharmacological effects of six polypore extracts were investigated in inflammatory DED-relevant *in vitro* assays.

Overall, the extracts proved to be safe in HCE-T cells and IHMGECs at concentrations up to 30 μg/mL. Only FF showed slight cytotoxic effects across the whole concentration range, and LO was considerably cytotoxic to HCE-T cells at a concentration of 30 μg/mL. Moreover, cytotoxicity was detected in THP-1 cells at lower concentrations (≥30 μg/mL) than in HCE-T cells and IHMGECs, especially in THP-1 cells treated with the *Ganoderma* species (GL and GT) and LO. Because most medicinal mushrooms contain polysaccharides (e.g. β-glucans) and steroids/triterpenoids (e.g. ergosterol and ganoderic acid), they are known not only for their potent antioxidant activity but also for their antiproliferative and proapoptotic effects. A number of studies have demonstrated such effects of *Ganoderma* species (Cheng et al., 2007; Gao et al., 2004; Ren et al., 2021). In addition, triterpenoids isolated from LO have demonstrated cytotoxicity against various cancer cell types (Feng and Yang, 2015; Han et al., 2020). In contrast, IO showed no cytotoxicity in HCE-T, iHMGEC, and THP-1 cells which is in line with previous studies (Chen et al., 2009; Shnyreva et al., 2018).

The ability of the studied fungal extracts to scavenge free radicals was concentration-dependent for all extracts and particularly pronounced for GT, GO, and IO. Similarly, GT, GO, and IO were observed to have strong effects in both UVB-exposed HCE-T cells and iHMGECs.

Among the tested extracts, GO exhibited the highest free radical scavenging ability. The characterization of the significant antioxidant activity of GO is based on a rich phenolic profile. Fumaric acid and protocatechuic acid were identified as the major constituents (Tel-Çayan, 2019). Furthermore, antioxidant and radical scavenging properties of polysaccharides of GT extracts are also known (Mau et al., 2005; Tseng et al., 2008). In *in vivo* studies on UV-induced oxidative stress, IO was shown to possess cytoprotective properties by reducing ROS and lipid peroxide levels (Yun et al., 2011). It was also found to have genoprotective properties by upregulating DNA repair genes (Eid et al., 2021). IO grows on birch trees, where it acquires a large quantity of betulin and betulinic acid (Ryvarden and Gilbertson, 1993). Both of which proved themselves through their potent antioxidant activity (Bai et al., 2012; Drenkhan et al., 2022; Géry et al., 2018).

Activation of NF-κB is crucial to initiate a wide range of transcription processes during inflammation. Numerous mushroom species contain secondary metabolites and polysaccharides that are known to reduce inflammatory mediators, including nitric oxide (NO), a variety of pro-inflammatory cytokines, and NF-κB (Amirullah et al., 2024; Taofiq et al., 2016). In this study, NF-κB expression in THP-1 cells was concentration-dependently reduced in all extracts. Only treatment with the IO extract had no effect on NF-κB expression. However, other studies showed a reduction in NF-κB level in murine macrophage cells (RAW 264.7 cells) after treatment with IO extract (Ma et al., 2013). Further studies are needed to investigate the impact of IO on other monocytic/macrophage cells, such as primary cells, to confirm this aspect.

Decreased NF-κB expression results in a reduction of the expression of the transcription factor and consequently in a decreased expression of various pro-inflammatory cytokines such as TNF-α, IL-1β, IL-6 and cyclooxygenase-2 (Liu et al., 2017). The inhibition of cytokine levels in THP-1 cells after treatment with the extracts observed in this study reflects this relationship, aside from the IO extracts. However, in addition to the NF-κB pathway, other factors also mediate the expression of pro-inflammatory factors, such as the nuclear factor of activated T cells (NFAT) or activator protein 1 (AP-1) (Lorentz et al., 2003).

Oxidative stress and eye irritation lead to cytokine-mediated (TNF-α, IL-6, and IL-8) inflammation. To determine the potential as a DED treatment, the extracts were therefore examined for their inhibitory properties against these cytokines. In HCE-T cells, GO showed the strongest inhibitory effects on cytokine levels at a concentration of 30 μg/mL. However, good effects were also achieved with 3 and 10 μg/mL GL. Furthermore, stronger effects and lower standard deviation errors were observed in HCE-T cells when testing lower concentrations than when testing high concentrations. Cytotoxic stress could likely occur during treatment with high concentrations (30 μg/mL) and affect cytokine secretion.

In comparison to HCE-T cells, the secretion of all the cytokines examined was significantly inhibited in THP-1 cells. The strongest effects using non-toxic concentrations (3 or 10 μg/mL) were observed for GT, followed by GL, IO, and GO. One study has shown that GT has bidirectional immunomodulatory effects on the secretion of cytokines by THP-1 cells, by downregulating TNF-α and IL-1 α when exposed to a high dose of LPS and phorbol myristate acetate (PMA) and the opposite occurs when exposed to a low dose of the stimulants (Gao et al., 2000). For IO, significantly higher TNF-α levels and IL-8 levels were detected at the highest concentration (30 μg/mL) compared to the positive control group. Another study also describes an induction of pro-inflammatory factors (TNF-α, IFN-γ, IL-1β, and IL-2) in human peripheral blood mononuclear cells (PBMCs) treated with IO extract, possibly due to the composition of heteropolysaccharides such as glucose, galactose and mannose (Xu et al., 2014).

The extracts of GO and GL showed significant inhibitory effects on the secretion of the observed pro-inflammatory cytokines in both HCE-T cells and THP-1 cells. In the present study, GO exhibited the strongest cytokine-inhibiting effect among the extracts tested. However, the anti-inflammatory properties of GO in cells have rarely been described in the literature to date. Hence, further studies are needed to explore its therapeutic properties in more detail.

GL, on the other hand, is one of the oldest and best-known traditional Chinese medicines, that has been demonstrated to have immunomodulating effects (Lin and Zhang, 2004) and to inhibit inflammatory mediators such as IL-6, IL-8, IL-10, and TNF-α in various cell types (Barbieri et al., 2017; Habijanic et al., 2015; Wu et al., 2019). Likewise, GL lowers IFN-γ, IL-1β, and TNF-α levels in THP-1 cells (Rathor et al., 2014) and reduces oxidative damage in corneal epithelial cells (Tsai et al., 2021). Thus, both GL and GO demonstrated significant anti-inflammatory potential.

In order to determine the impact of the extracts on the cell-tight junction, the TEER value was determined. Both GL and GO at a concentration of 30 μg/mL decreased the TEER values after 48 h of incubation. Since epithelial cell viability correlates with high TEER readings (Konsoula and Barile, 2005; Reichl, 2008), cytotoxicity caused by long-term exposure to the extracts could explain the results. On the other hand, LO, which was found to be the most cytotoxic extract in this study, has shown higher TEER values than the negative control with low permeability of the sodium fluorescein. Since the wound healing experiment showed no stimulatory effect of the extracts on cell proliferation, this is unlikely to be the cause. Thus, the morphology of the membrane proteins such as ZO-1 and occludin or the cell size could be affected by LO. Further studies are needed to confirm this assumption.

The three primary ingredients of the tear film layer are lipids, water, and mucin. Meibomian gland cells release lipids that coat the outermost layer of the cornea, shielding it from the environment and preventing evaporation of the tear fluid. It has been demonstrated that meibomian gland dysfunction is a common factor of DED development, where reduced lipid secretion disrupts the tear film homeostasis. This results in ocular irritation, hyperosmolarity and inflammation (Baudouin et al., 2016). It has been discovered that monounsaturated oleic fatty acid is present at reduced concentrations in patients with meibomian dysfunction (Shine and McCulley, 2000). Stearoyl-CoA desaturase and fatty acid synthase are some the important lipid synthesis markers that have been found to increase meibomian lipid production when expressed (Liu et al., 2011). Our test samples may lack essential compounds to upregulate the aforementioned related lipid genes to stimulate lipid production. On the other hand, a study has shown that ergosterol peroxide, a steroid commonly found in mushrooms and extracted from GL inhibited lipid droplet production, triglycerides, and differentiation of 3T3-L1 adipocytes (Jeong and Park, 2020). Ergosterol peroxide has also been found in FF, GO and IO (Kahlos, 1996; Ma et al., 2013; Rösecke and König, 2000) but was not detected in the herein investigated extracts. Moreover, in this study, no impacts on the amount of secreted lipid droplets were detected in treated IHMGECs, an increase in the size of secreted lipid droplets was observed when treated with GT extract.

Finally, the influence of the extracts on the tight junction connections of HCE-T cells was investigated. Both GL and GO disrupted the tight junctions of HCE-T cells after 24 h of treatment, while LO strengthened the cells’ tight junctions and consequently the barrier function of the cells, which was also confirmed by the low paracellular permeability of the sodium fluorescein tracer in the basolateral chamber of the transwell. The main group of constituents of LO consists of triterpenoids of the lanostane type (Grienke et al., 2014). These triterpenoids have been shown to induce neuromuscular blockade, which causes botox-like effects (Santana et al., 2011). Botox has been proposed for the treatment of DED because of its ability to reduce lacrimal drainage (Sahlin and Chen, 1996). However, in practice, no noticeable improvement has been observed in DED patients treated with botox (Horwath-Winter et al., 2003).

## 5 Conclusion

In this study, we investigated the mycochemical compositions and therapeutic potential of six ethanolic fungal extracts (FF, GL, GT, GO, IO, and LO) for the treatment of DED using a set of *in vitro* assays. The mycochemical analysis revealed that except for FF, all extracts contain lanostane triterpenes as main secondary metabolites. Interestingly, among the triterpene containing extracts a broad chemical diversity of this structure class was observed. This is reflected by modifications such as oxygenation and esterification. This might explain the different biological properties observed. In general, the extracts were shown to be effective in reducing oxidative stress, one of the relevant factors in the production of ROS. Pronounced antioxidant activities in corneal cells were observed for GT, GO, and IO. Cytokine secretion by THP-1 cells was significantly inhibited in a concentration-dependent manner by all extracts except for IO. Nevertheless, the anti-inflammatory investigations of the extracts revealed that in all HCE-T, iHMGEC, and THP-1 cells, GO and GL had significant effects in reducing the aetiology associated with DED. Fungal extracts in particular those of GO and GL warrant further investigations to clarify their specific therapeutic benefits and mechanisms of action and to identify their bioactive components in relation to DED. They showed to possess the potential for controlling inflammatory response and oxidative stress, which paves the way for their application in modern therapeutic contexts, such as in managing dry eye disease (DED).

## Data Availability

The raw data supporting the conclusions of this article will be made available by the authors, without undue reservation.

## Abbreviations

DED, dry eye disease; DPPH, 2,2-diphenyl-1-picrylhydrazyl; EGF, epidermal growth factor; FF, *F. fomentarius*; GO, *G. odoratum*; GL, *G. lucidum*; GT, *G. tsugae*; HBSS, hank’s balanced Salt solution; HCE-T, human corneal epithelial cell-transformed; H2DCFDA, 2′,7′-dichlorodihydrofluorescein diacetate; IHMGECs, Immortalized human meibomian gland epithelial cells; IL, interleukin; NF-κB, nuclear factor-kappa B; IO, *I. Obliquus*; LO, *L. officinalis*; PBS, phosphate-buffered saline; THP-1, human leukemia monocytic cell line; UVB, ultraviolet B; WST-1, water-soluble tetrazolium salt.
