# Supplementary material for: Evaluation of in vitro pharmacological activities of medicinal mushrooms in the context of dry eye disease
Source: Front Pharmacol. 2025 Mar 5;16:1557359. doi: 10.3389/fphar.2025.1557359 (PMC11920184; doi:10.3389/fphar.2025.1557359)
Supplement: Supplementary file 1 [file DataSheet1.docx]

**Evaluation of *in vitro* pharmacological activities of medicinal mushrooms in the context of dry eye disease**

**Alexander Areesanan^1^**, Andreas Wasilewicz^2^, Sven Nicolay^1^, Ulrike Grienke^2^, Amy Marisa Zimmermann-Klemd^1^, Judith M. Rollinger^2^, Carsten Gründemann^1*^

1 Translational Complementary Medicine, Department of Pharmaceutical Sciences, University of Basel, Switzerland

2 Division of Pharmacognosy, Department of Pharmaceutical Sciences, Faculty of Life Sciences, University of Vienna, Austria

* Corresponding author

* Corresponding author

Prof. Dr. Carsten Gründemann

Campus Rosental – Mattenstrasse 22, 4058 Basel, Switzerland

Tel: +41 61 207 61 84

Mail: carsten.gruendemann@unibas.ch

**Table ST1.** Traditional use of six medicinal polypore mushrooms.

| **Species name** | **Common names** | **Global distribution** | **Traditional use** |
| --- | --- | --- | --- |
| *Fomes*  *fomentarius* (L.) Fr. | Tinder polypore, tinder conk, amadou, hoof fungus | Africa, Asia, North America, and Europe | Igniter, insect repellent, spiritual reasons, haemostatic dressings, dysmenorrhea, haemorrhoids and bladder disorders, pain |
| *Ganoderma lucidum* (Fr.) P. Karst. | Reishi, Lingzhi | Asia and North America | Antitumour, spleen and stomach nourishment, improve eyesight, strengthen muscles and bones, promote health and longevity |
| *Ganoderma tsugae* Murrill | hemlock varnish shelf | Asia and North America | No use reported |
| *Gloeophyllum odoratum* (Wulfen) Imazeki | Anise mazegill and brown rot fungus | Europe | No use reported |
| *Inonotus obliquus* (Fr.) Pilát | Chaga | Asia, Europe, and North America | Anti-parasitic, anti-tuberculosis, anti-inflammatory, and gastrointestinal diseases |
| *Laricifomes officinalis* (Vill.) Kotl. & Pouzar | Agarikon, eburiko, quinine conk | Europe, North America | Gastric cancer, rheumatism, tuberculosis, pneumonia, cough, and asthma |

**Table ST2.** Fungal materials, source and voucher specimens of investigated fungal extracts.

| **Species** | **Family** | **Organ** | **Source** | **Voucher specimen** |
| --- | --- | --- | --- | --- |
| *Fomes fometarius* (L.) Fr | Polyporaceae | fruit body | Neue Magdeburger Hütte (1300 m), Zirl, Austria (grown on beech) | FomfomE0019 |
| *Ganoderma lucidum* (Fr.) P. Karst. | Ganodermataceae | fruit body | Plantasia GmbH, Oberndorf bei Salzburg, Austria | JR-20150313-B1 |
| *Ganoderma tsugae* Murill | Ganodermataceae | fruit body | MRCA Mushroom Research Center Austria GmbH, Innsbruck, Austria | JR-20130120-A2 |
| *Gloeophyllum odoratum* (Wulfen) Imazeki | Gloeophyllaceae | fruit body | Oberperfuss, Austria (grown on spruce) | GloodoE0054 |
| *Inonotus obliquus* (Fr.) Pilát | Hymenochaetaceae | fruit body | Finnland, 1998 | UP-20121212-A1 |
| *Laricifomes officinalis* (Vill.) Kotl. & Pouzar | Laricifomitaceae | fruit body | MRCA Mushroom Research Center Austria GmbH, Innsbruck, Austria | JR-20201027-A4 |

**
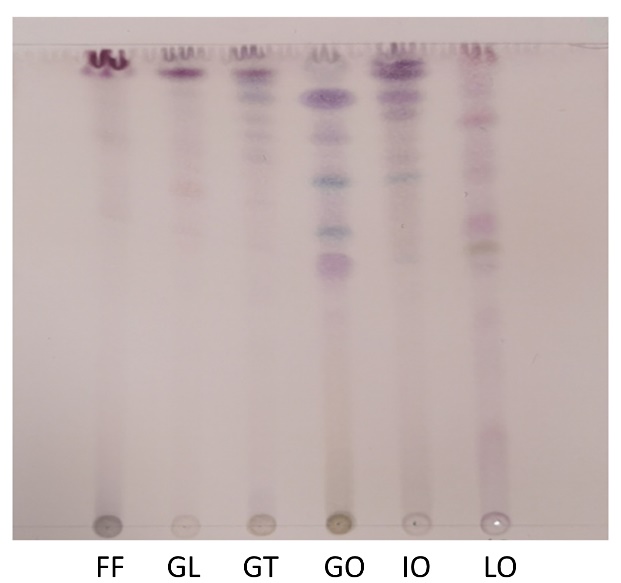
**

**Figure SF1**. TLC chromatogram of mycochemically analysed fungal extracts. Detection at visible light after derivatization with vanillin (1%)/sulfuric acid (5%).
